# KOH-modified bamboo charcoal loaded with α-FeOOH for efficient adsorption of copper and fluoride ions from aqueous solution†

**DOI:** 10.1039/d3ra05315f

**Published:** 2023-10-16

**Authors:** Wei Yang, Lei Zhang, Meng Li, Ting Zhang, Yue Liu, Juan Liu

**Affiliations:** a School of Environmental Science and Engineering, Hubei Polytechnic University Huangshi 435003 Hubei China yw198909@163.com; b MWR Standard & Quality Control Research Institute Hangzhou 310024 Zhejiang China; c School of Civil Engineering and Architecture, Wuhan University of Technology Wuhan 430070 Hubei China

## Abstract

In this work, bamboo charcoal (BC) is prepared by pyrolysis of bamboo. Then, KOH modification and surface deposition of Goethite (α-FeOOH) are performed to obtain a new KOH-modified BC loaded with α-FeOOH (FKBC) adsorbent for copper (Cu^2+^) and fluoride (F^−^) ion adsorption from aqueous solution. Surface morphology and physiochemical properties of the prepared adsorbent are characterized by scanning electron microscopy-energy dispersive spectrometer, X-ray diffraction, and N_2_ adsorption–desorption. The effect of pH, contact time, adsorbent dosage, and initial concentration on Cu^2+^ and F^−^ adsorption is also investigated. In addition, adsorption kinetics and isotherms are fitted to pseudo-second-order kinetics and Langmuir model, respectively. Thermodynamic parameters suggest that the adsorption process is spontaneous and endothermic. The adsorption mechanism is further characterized by Fourier transform infrared spectroscopy and X-ray photoelectron spectroscopy. The Cu^2+^ absorption mainly occurs through ion exchange, coordination reactions, and surface precipitation, while the F^−^ adsorption mainly occurs *via* ion exchange and hydrogen bonding. The selective adsorption experiments reveal that FKBC has good selectivity for Cu^2+^ and F^−^. The adsorption–desorption experimental results indicate that FKBC can be reused for Cu^2+^ and F^−^ adsorption after regeneration. Results indicate that FKBC can be a promising adsorbent for Cu^2+^ and F^−^ removal from aqueous solutions.

## Abstract 2

A novel KOH-modified bamboo charcoal loaded with α-FeOOH (FKBC) was successfully synthesized and used for highly efficient Cu^2+^ and F^−^ adsorption.

## Graphical abstract

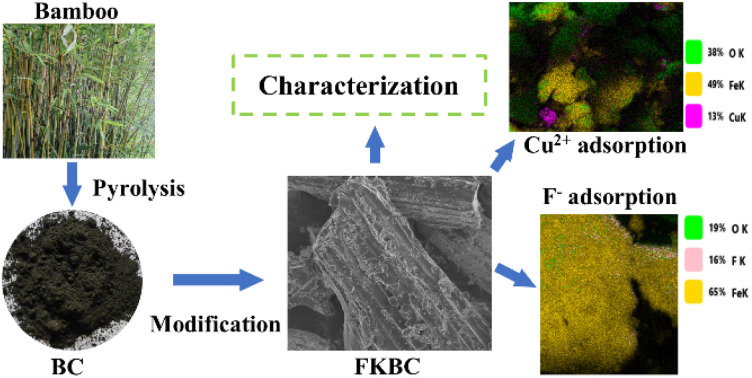

## 1. Introduction

With the development of the global electronic information industry, the demand for printed circuit boards (PCBs) and semiconductor chips has rapidly increased. A large amount of wastewater containing copper (Cu^2+^) and fluoride (F^−^) ions has been generated during pickling of copper-clad substrates on PCBs and the etching and cleaning process of semiconductor chip surfaces, respectively.^1,2^ Discharging Cu^2+^ and F^−^ into water and soil seriously impacts the ecological environment system and human body health through bioaccumulation and the food chain.^3,4^ Therefore, finding an effective treatment method to remove Cu^2+^ and F^−^ from wastewater is necessary.

Currently, the main methods for Cu^2+^ removal include adsorption,^5,6^ ion exchange,^7^ coagulation,^8^ chemical precipitation,^9^ membrane capacitance deionization,^10^ and reverse osmosis.^11^ Similarly, adsorption,^12,13^ ion exchange, electrodialysis, and reverse osmosis^14^ have also been used for F^−^-removal from wastewater. Among these, adsorption is the most effective and popular method due to the advantages of low treatment cost, flexible treatment process, and recyclable characteristics, which are suitable for treating various wastewater concentrations.^15^ Compared with adsorption, other treatment methods have shortcomings, such as high treatment costs, generation of liquid sludge, and secondary pollution.^16^

Adsorption cost is an important consideration for using adsorption technology to remove harmful substances from wastewater, and the latest research trend mainly focuses on low-cost biochar adsorbents.^17,18^ Carbon-rich biochar adsorbents from different sources, including biological sludge,^19^ wood chips,^20^ plant residues,^21^ and agricultural waste,^22,23^ bamboo^24–26^ generally exhibit large porosity, specific surface area, and a variety of functional groups of biochar surface. Compared with other raw materials for preparing biochar, bamboo is a rich renewable biomass with fast growth, widespread planting, and low cost; thus, it is a perfect raw material for biochar production.^27^

Although biochar has a certain adsorption effect on harmful substances, further biochar modification is needed to compensate for the insufficient adsorption capacity.^28^ Some researchers have modified biochar through chemical methods to change its microporous structure, functional groups, specific surface area, and adsorption binding sites. Wang *et al.*^29^ synthesized alkali-modified biochar from bamboo impregnation with KOH *via* pyrolysis and used it for Cu^2+^ removal with higher adsorption capacity than the original bamboo charcoal. Thakur *et al.*^30^ used pine biomass mixed with KOH by high-temperature pyrolysis to synthesize alkali-modified pine biochar, achieving better adsorption for F^−^. Depositing metal compounds on biochar surfaces can increase the binding sites and enhance adsorption capacity.^31^ Liang *et al.*^32^ reported Cu^2+^ adsorption by loading Ca–Fe layered double hydroxide on corn straw biochar surface. The adsorption capacity for Cu^2+^ was increased twice compared to the original corn straw biochar. Pillai *et al.*^33^ prepared a rice husk biochar loaded with iron oxyhydroxide, demonstrating an excellent F-adsorption capacity.

In particular, metal hydroxide minerals have been extensively reported as good adsorbents.^34^ Goethite (α-FeOOH) is widely present in nature and can be easily synthesized in the laboratory. It has been increasingly used as an adsorption material due to its strong affinity, special structure, number of binding sites, environmental friendliness, and low cost.^35,36^ Literature has confirmed that α-FeOOH can effectively adsorb Cu^2+^ and has strong affinity and adsorption ability for F^−^ due to its reactive functional groups.^37,38^ However, α-FeOOH is prone to aggregation, limiting its practical applications.^39,40^ Therefore, depositing α-FeOOH onto biochar to improve its dispersion benefits its widespread utilization in environmental applications.

Based on this information, we anticipate alkali modification of bamboo charcoal (BC) and α-FeOOH surface deposition can produce a potential adsorption material for Cu^2+^ and F^−^ removal. Therefore, this study prepares a novel KOH-modified BC loaded with α-FeOOH (FKBC). The physicochemical properties of FKBC have been investigated. The effect of pH, contact time, adsorbent dosage, initial concentration on Cu^2+^ and F^−^ adsorption, adsorption kinetics, isotherms, thermodynamics, and adsorption mechanism are studied. Finally, the adsorption selectivity and reusability of FKBC for Cu^2+^ and F^−^ are also evaluated. Nevertheless, no investigation has been previously reported on FKBC for simultaneous Cu^2+^ and F^−^ removal.

## 2. Materials and methods

### 2.1 Materials and reagents

The bamboo used in the experiment was collected from Hubei Polytechnic University, Hubei Province, China. KOH, HNO_3_, Cu(NO_3_)_2_, KF, Fe(NO_3_)_3_, NaCl, K_2_SO_4_, KNO_3_, and NH_4_Cl were purchased from China National Pharmaceutical Group Chemical Reagents Co., Ltd. NiSO_4_ and CdSO_4_ were obtained from Aladdin Reagent Co., Ltd. All chemical reagents used in these experiments were of analytical grade and were used without further pretreatment. Cu^2+^ and F^−^ working solutions were prepared by dissolving Cu(NO_3_)_2_ and KF in deionized water, respectively. pH of Cu^2+^ and F^−^ working solutions was adjusted by KOH (1 mol L^−1^) and HNO_3_ (1 mol L^−1^).

### 2.2 Adsorbent preparation

BC adsorbent was prepared according to previous work.^29,41^ First, 100 g fresh bamboo was cut into small pieces, washed three times with deionized water, and dried in an oven at 353 K for 6 h. The BC was prepared in a resistance tube by calcinating the bamboo pieces at 923 K for 1.5 h under an N_2_ atmosphere of 100 mL min^−1^ flow rate. The obtained BC was then ground and sieved at 100 microns.

The preparation procedure of FKBC was referred to in the previous paper.^31,40^ 20 g BC powder was added to a 200 mL KOH solution (2 mol L^−1^) and impregnated at 333 K for 12 h in a water bath shaker at 120 rpm. Then, 100 mL Fe(NO_3_)_3_ solution (1 mol L^−1^) was slowly added to the BC-KOH mixture to form a precipitate, aged in an oscillating water bath at 333 K for 48 h before natural precipitation. The precipitate was washed with deionized water until the pH was neutral. The product was dried at 333 K for 6 h, ground, and sieved at 100 microns.

### 2.3 Characterization of FKBC

The surface and elemental composition of FKBC were analyzed by scanning electron microscopy (SEM, SU8220, Japan) combined with an energy dispersive spectrometer (EDS, K-Alpha, UK), respectively. The mineral composition was determined by X-ray diffraction analysis (XRD, D/max-3B, Japan). The adsorbent's specific surface area and pore volume were obtained by the N_2_ adsorption–desorption method at 77 K (Micromeritics TriStar II 3020, USA). The surface potential of the adsorbent was measured by the pH drift method. Fourier transform infrared spectroscopy (FTIR) was used to characterize the surface functionalities of the adsorbent before and after adsorption (Nicolet 6700, USA). X-ray photoelectron spectroscopy (XPS) was used to study the surface chemical state of the adsorbent before and after adsorption (ESCALAB250Xi, USA).

### 2.4 Batch adsorption experiments

Batch adsorption experiments were conducted in a 200 mL conical flask at a shaking speed of 120 rpm. At the end of each experiment, a 2 mL solution sample was filtered with a 0.22 μm membrane. The residual Cu^2+^ and F^−^ concentrations in the filtrate were determined by atomic absorption spectroscopy (AAS) and ion chromatography (IC), respectively.

Detailed experimental conditions for batch adsorption, such as pH, contact time, adsorbent dosage, Cu^2+^, F^−^ initial concentration, kinetics, isotherms, and thermodynamics, are provided in ESI (Table S1†). Parameter details and coexisting ions are discussed in selective adsorption experiments (Table S2†). The adsorption efficiency (*R*%), adsorption capacity (*Q*_*t*_, mg g^−1^) at time *t*, and equilibrium adsorption capacity (*Q*_e_, mg g^−1^) of FKBC for Cu^2+^ or F^−^ was calculated by the following equations:^42^1
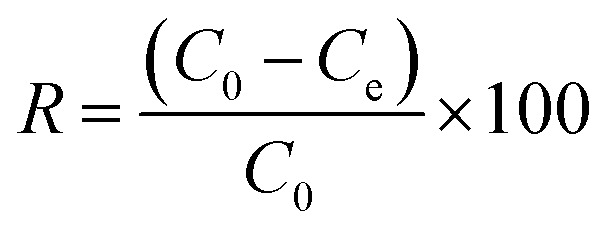
2
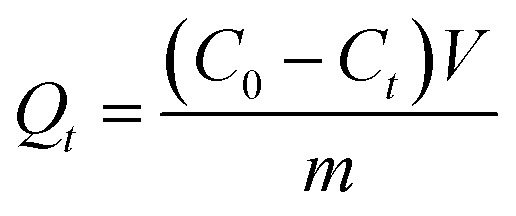
3
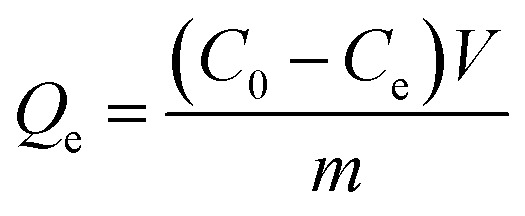
where *C*_0_ is the Cu^2+^ or F^−^ initial concentration (mg L^−1^), *C*_e_ is the Cu^2+^ or F^−^ concentration at adsorption equilibrium (mg L^−1^), *C*_*t*_ is Cu^2+^ or F^−^ concentration over time (mg L^−1^), *V* is the solution volume (L), and *m* is the amount of FKBC (g) used in the experiments.

### 2.5 Recycling experiments

Several adsorption–desorption cycle experiments were conducted under 120 rpm to examine the reusability of the adsorbent. Detailed experimental conditions are shown in Table S3.† After adsorption, the Cu^2+^ or F^−^-loaded FKBC was filtered with 0.22 μm membrane and regenerated with 100 mL HNO_3_ (1 mol L^−1^) and KOH (1 mol L^−1^) for 1 h, respectively. Then, the desorbed adsorbent was washed with deionized water several times and dried for the next adsorption experiments.

### 2.6 Statistics analysis

In this study, all adsorption experiments are conducted in triplicate to ensure consistency. The experiment results are calculated as mean ± standard error. Data are analyzed by one-way analysis of variance (ANOVA) and Student–Newman–Keuls (SNK) *post hoc* test comparisons using the IBM SPSS Statistics 21.0 software. The statistical significance is set at a level of *p* < 0.05. The kinetic, isotherm, and thermodynamic data fitting are performed using the Origin 2021 software.

## 3. Results and discussion

### 3.1 Characterization

**Fig. 1:**
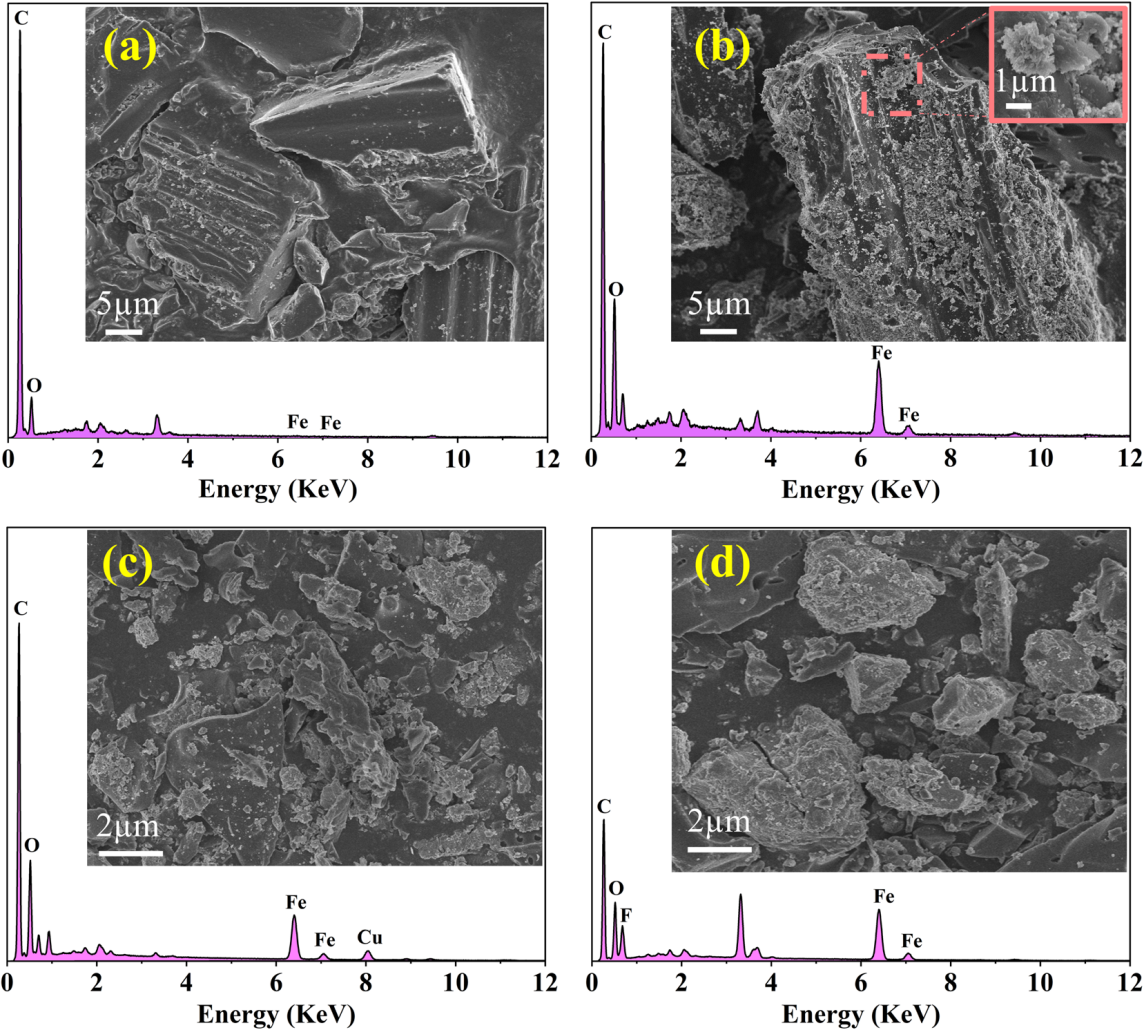
SEM-EDS images of the BC (a), FKBC before (b), and FKBC after (c) Cu^2+^ and (d) F^−^ adsorption.

Fig. 1(a) and (b) show the SEM-EDS images of BC and FKBC. The BC surface is relatively smooth without an obvious fold structure. In contrast, the FKBC surface is rough, attaching much sediment with micropores. EDS analysis reveals the absence of Fe in BC; however, the Fe content in FKBC is up to 9.8%, while the O content increases from 10.53% in BC to 19.66% in FKBC (Table 1). In addition, the EDS mapping results show that Fe and O elements are almost concentrated on the FKBC surface sediments (Fig. S1(a–c) and S2(a–c)†). These results preliminarily confirm the alkali modification on the biochar surface and α-FeOOH loading. SEM images in Fig. 1(c) and (d) depict that after reuse of FKBC for Cu^2+^ and F^−^ adsorption, the surface is rough with a large amount of sediment, suggesting that the morphology of FKBC does not change after reuse. EDS element analysis also confirms the successful adsorption of Cu^2+^ and F^−^.

**Table 1:** The physiochemical properties of BC and FKBC^a^

Samples	Surface atomic relative content (wt%)						*S*_BET_ (m^2^ g^−1^)	*V*_tot_ (cc g^−1^)	Pore diameter (nm)
	C	O	Fe	Cu	F	Others			
BC	83.14	10.53	—	—	—	6.33	1.25	0.034	6.54
FKBC	67.53	19.66	9.80	—	—	3.01	19.79	0.126	3.54

a The others are Si, H, and K; *S*_BET_: specific surface area; *V*_tot_: total pore volume.

**Fig. 2:**
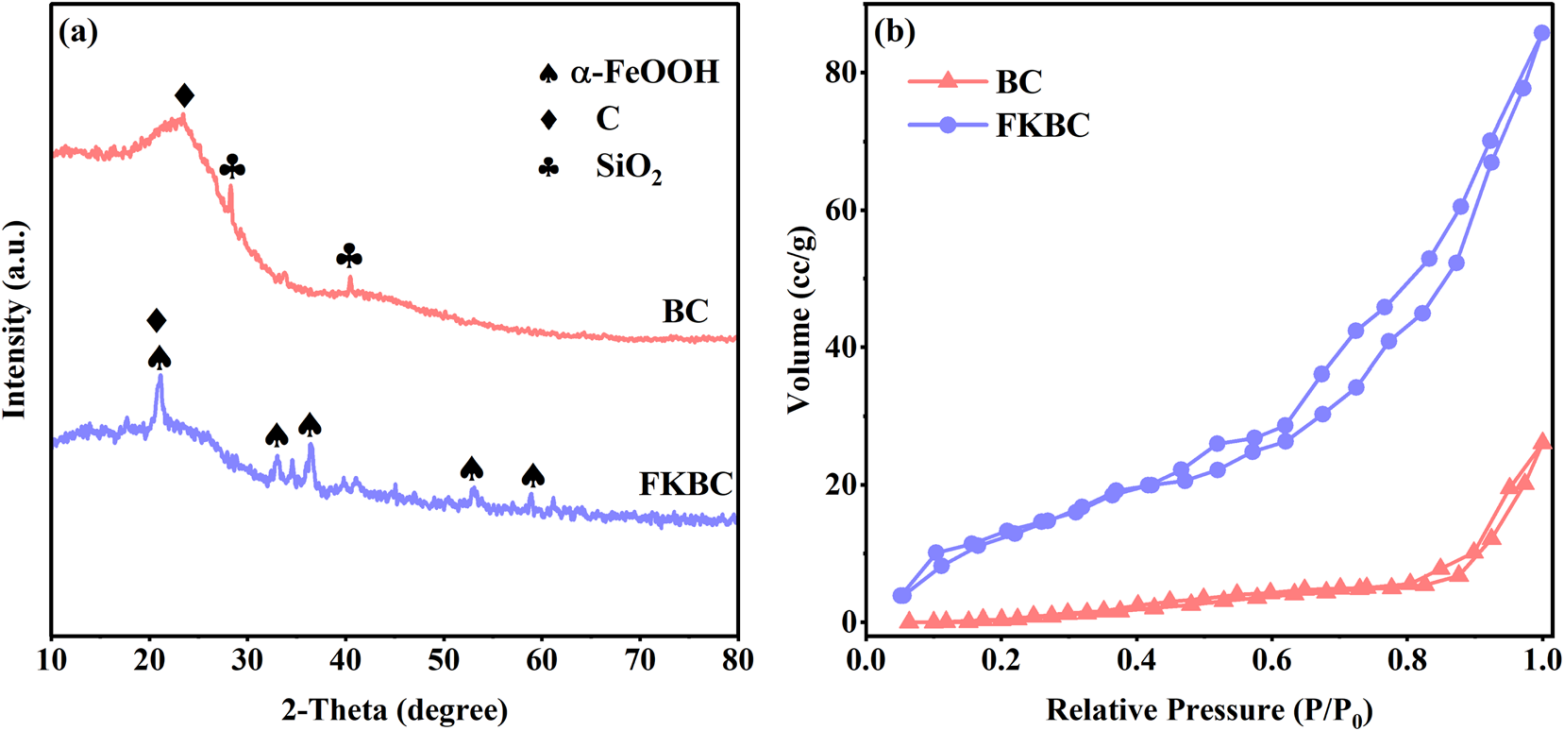
(a) XRD patterns and (b) N_2_ adsorption–desorption isotherms of BC and FKBC.

XRD patterns of BC (Fig. 2(a)) show that a broad and low-intensity peak appears at 2*θ* = 23°, attributed to aromatization and graphitization of carbonaceous organic matter.^43^ Peaks at 28.40° and 40.48° are assigned to (111) and (210) planes of SiO_2_ (PDF 76-0935). These SiO_2_ impurities are present in biochar pores and are unfavorable for active adsorption site exposure.^44^ The XRD spectrum of FKBC shows that the SiO_2_ characteristic peaks disappear, suggesting that the biochar surface impurities are eliminated after alkali modification, promoting the surface pores formation.^45^ The characteristic peaks at 21.24°, 33.24°, 36.66°, 53.24°, and 59.00° belong to (110), (130), (111), (221), and (151) of α-FeOOH (PDF81-0464), suggesting that α-FeOOH is successfully loaded on biochar surface.^38^

Fig. 2(b) shows the N_2_ adsorption–desorption isotherms of BC and FKBC. The N_2_ adsorption–desorption curve of FKBC indicates obvious hysteresis loops at high pressure and features of the type-IV model, demonstrating the formation of mesoporous structures.^46^Table 1 shows that the FKBC BET surface area is 19.79 m^2^ g^−1^, and the total pore volume is 0.126 cc g^−1^, 14.8 times and 2.7 times higher than BC, respectively. The FKBC pore diameter is 3.54 nm, smaller than that of BC. Therefore, FKBC has a larger specific surface area, pore volume, and smaller pore size, offering more adsorption sites and playing a vital role in Cu^2+^ and F^−^ adsorption from the aqueous phase.

### 3.2 Effect of the parameters on Cu^2+^ and F^−^ adsorption

#### 3.2.1 Effect of pH

The solution pH always dominates adsorption, influencing the chemical structure and adsorption sites and affecting the existing forms of Cu^2+^ and F^−^.^47^ Cu^2+^ mainly exists in the solution when the pH is between 1 and 5. However, light blue copper hydroxide precipitation appears when the pH exceeds 6.^4^ When the pH of the F^−^ solution is less than 2, they are mainly in the form of HF; when the pH is higher than 5, they are in the form of F^−^.^48^ Therefore, determining the optimum pH is necessary at the beginning of the Cu^2+^ and F^−^ adsorption study.

**Fig. 3:**
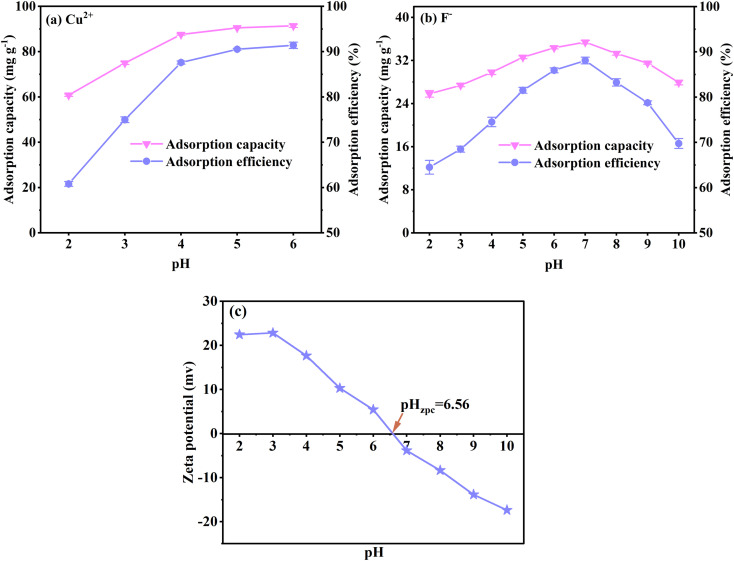
Effect of pH on Cu^2+^ (a) and F^−^ (b) adsorption by FKBC; (c) the zeta potential of FKBC.

Fig. 3(a) shows that the Cu^2+^ adsorption capacity and efficiency rapidly increase between pH 2 to 5 and slightly increase from pH 5 to 6. For F^−^ adsorption, the capacity and efficiency improve significantly between pH 2 to 7 and decrease from pH 7 to 10 (Fig. 3(b)). The results illustrate that the solution's excessive or low pH influences the Cu^2+^ and F^−^ adsorption. To explain this phenomenon, the surface potential of FKBC is measured. Fig. 3(c) shows that the zero-point charge (pH_zpc_) of FKBC is 6.56. The FKBC surface is positively charged when the pH of the solution is less than pH_zpc_, resulting in electrostatic repulsion, hindering the Cu^2+^ diffusion to the adsorbent surface.^49^ In addition, H^+^ in the solution combines with F^−^ to form HF, limiting F^−^ adsorption by FKBC. The surface is negatively charged when the pH > pH_zpc_. With the OH^−^ on the FKBC surface increasing, the competitive adsorption between F^−^ and OH^−^ strengthens. Also, the electrostatic repulsion for F^−^ increases, which is not conducive to F-adsorption. However, the precipitated flocs appear in Cu^2+^ solution at pH > 5, indicating that the enhanced removal is mainly attributed to precipitation, distinguished from adsorption and avoided.^6^ Therefore, for subsequent adsorption experiments, the pH of the Cu^2+^ and F^−^ working solution is set at 5 and 7, respectively.

#### 3.2.2 Effect of contact time

**Fig. 4:**
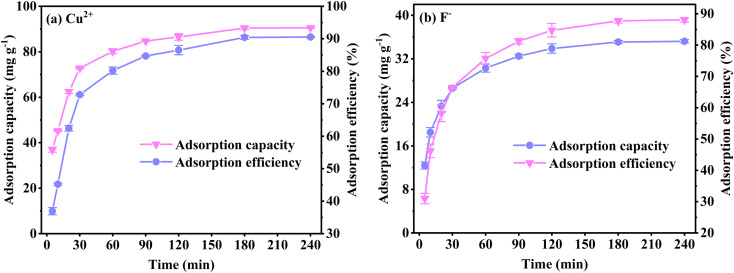
Effect of contact time on Cu^2+^ (a) and F^−^ (b) adsorption by FKBC.

Fig. 4 shows the effect of contact time on Cu^2+^ and F^−^ adsorption. During the first 30 minutes, the Cu^2+^ and F^−^ adsorption by FKBC is rapid. However, the adsorption rate decreases when the contact time exceeds 30 minutes. At the beginning of the adsorption process, Cu^2+^, and F^−^ are quickly adsorbed onto the adsorbent surface through mass transfer; as the reaction proceeds, most of the surface sites of FKBC are occupied; therefore, other Cu^2+^ and F^−^ diffuse into the porous structure, which is a relatively slow process compared to surface adsorption. Also, the Cu^2+^ and F^−^ adsorption approaches equilibrium at 90 and 120 minutes, respectively. However, the contribution of extended contact time to the adsorption effect is insignificant. Finally, when the contact time reaches 180–240 minutes, the adsorption efficiency and capacity for Cu^2+^ and F^−^ are 90.5%, 90.5 mg g^−1^ and 88.0%, 35.2 mg g^−1^, respectively, indicating that almost all adsorption sites and pores are occupied, bringing the adsorption reaction to an equilibrium state. Based on the results, the contact time of 240 minutes is appropriate.

#### 3.2.3 Effect of adsorbent dosage

**Fig. 5:**
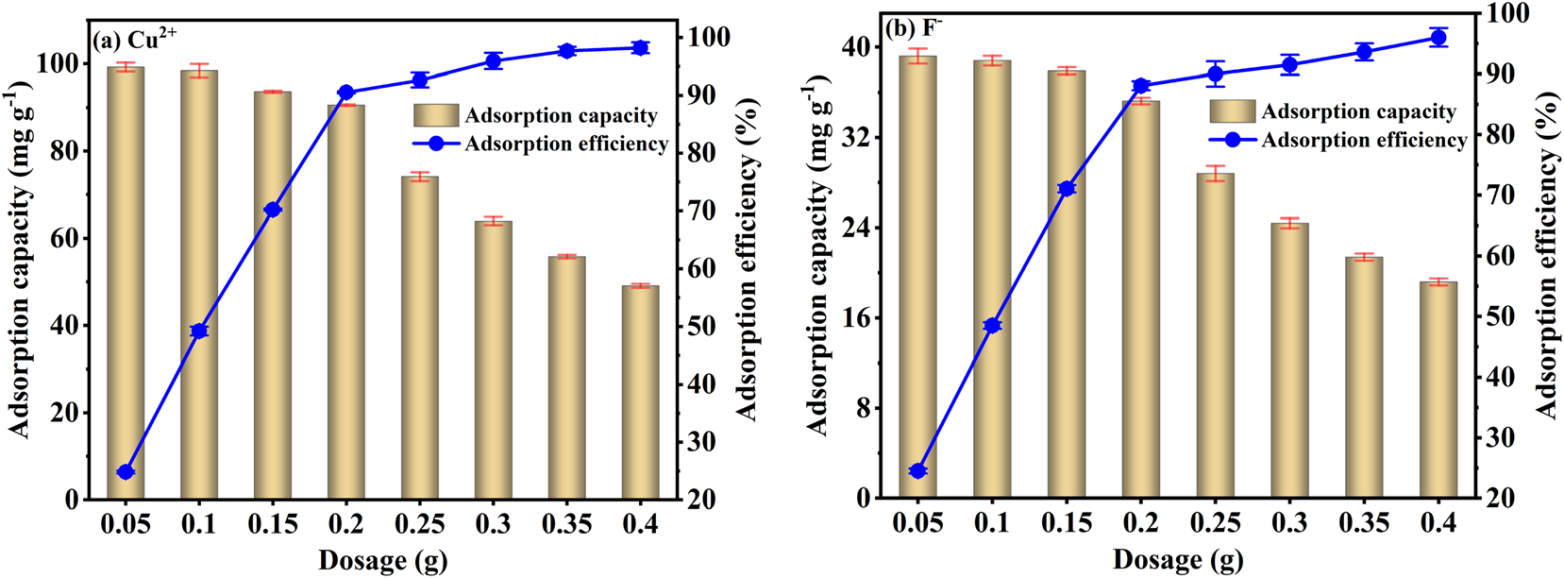
Effect of adsorbent dosage on Cu^2+^ (a) and F^−^ (b) adsorption by FKBC.

Fig. 5 shows the effect of FKBC dosage on Cu^2+^ and F^−^ adsorption. With the increase in FKBC dosage at constant Cu^2+^ and F^−^ content, the adsorption capacity gradually decreases while the adsorption efficiency gradually increases. This is due to an increase in FKBC content increases the total number of adsorption sites, thereby improving the adsorption efficiency. However, when the FKBC dosage reaches 0.2 g, the adsorption efficiency tends to stabilize. With further increased adsorbent amount, the ion concentration per unit surface area of FKBC decreases, making it difficult to improve adsorption efficiency. Moreover, as adsorbent dosage increases, the amount of adsorbed ions per unit surface area decreases,^50^ thus decreasing FKBC adsorption capacity for Cu^2+^ and F^−^.

#### 3.2.4 Effect of initial concentration

**Fig. 6:**
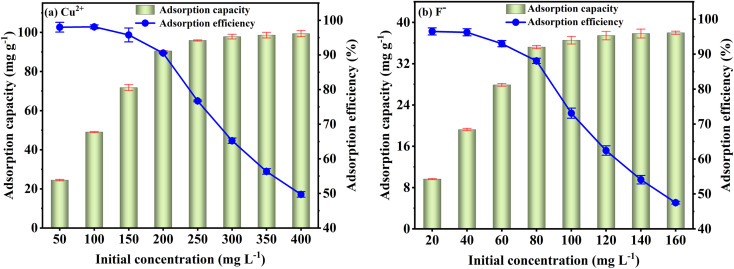
Effect of initial concentration on Cu^2+^ (a) and F^−^ (b) adsorption by FKBC.

Fig. 6 shows the FKBC adsorption efficiency for different Cu^2+^ and F^−^ initial concentrations. For a fixed FKBC dosage, when the Cu^2+^ and F^−^ initial concentrations are from 50 to 200 mg L^−1^ and 20 to 80 mg L^−1^, respectively, their adsorption efficiency decreases; however, the adsorption capacity increases rapidly. The main reason is that the Cu^2+^ and F^−^ initial concentrations are in lower range, many surface sites are available for ions' adsorption, increasing the adsorption capacity. Subsequently, with the same number of adsorption sites, the adsorption efficiency rapidly decreases as the initial concentration increases due to the saturation of adsorption capacity. Thus, the adsorption efficiency can be improved with an increasing adsorbent dosage for higher Cu^2+^ and F^−^ initial concentrations.^50^

### 3.3 Adsorption kinetics

The adsorption kinetics are analyzed to investigate the Cu^2+^ and F^−^ adsorption rate and the mechanism by FKBC. This study simulated the experimental adsorption data related to Cu^2+^ and F^−^ using pseudo-first-order, pseudo-second-order, and intraparticle-diffusion models.

Pseudo-first-order (eqn (4)), pseudo-second-order (eqn (5)), and intraparticle-diffusion models (eqn (6)) are shown below.

4 *Q*_*t*_ = *Q*_e_(1 − e^−*k*_1_*t*^)

5
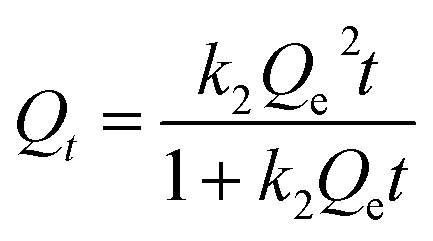

6 *Q*_*t*_ = *k*_ip_*t*^0.5^ + *C*

where *k*_1_ (min^−1^), *k*_2_ (g mg^−1^ min^−1^), and *k*_ip_ (mg g^−1^ min^−0.5^) are the rate constants related to pseudo-first-order, pseudo-second-order, and intraparticle-diffusion models, respectively, *c* (mg g^−1^) is the intercept of the intraparticle-diffusion model.

**Fig. 7:**
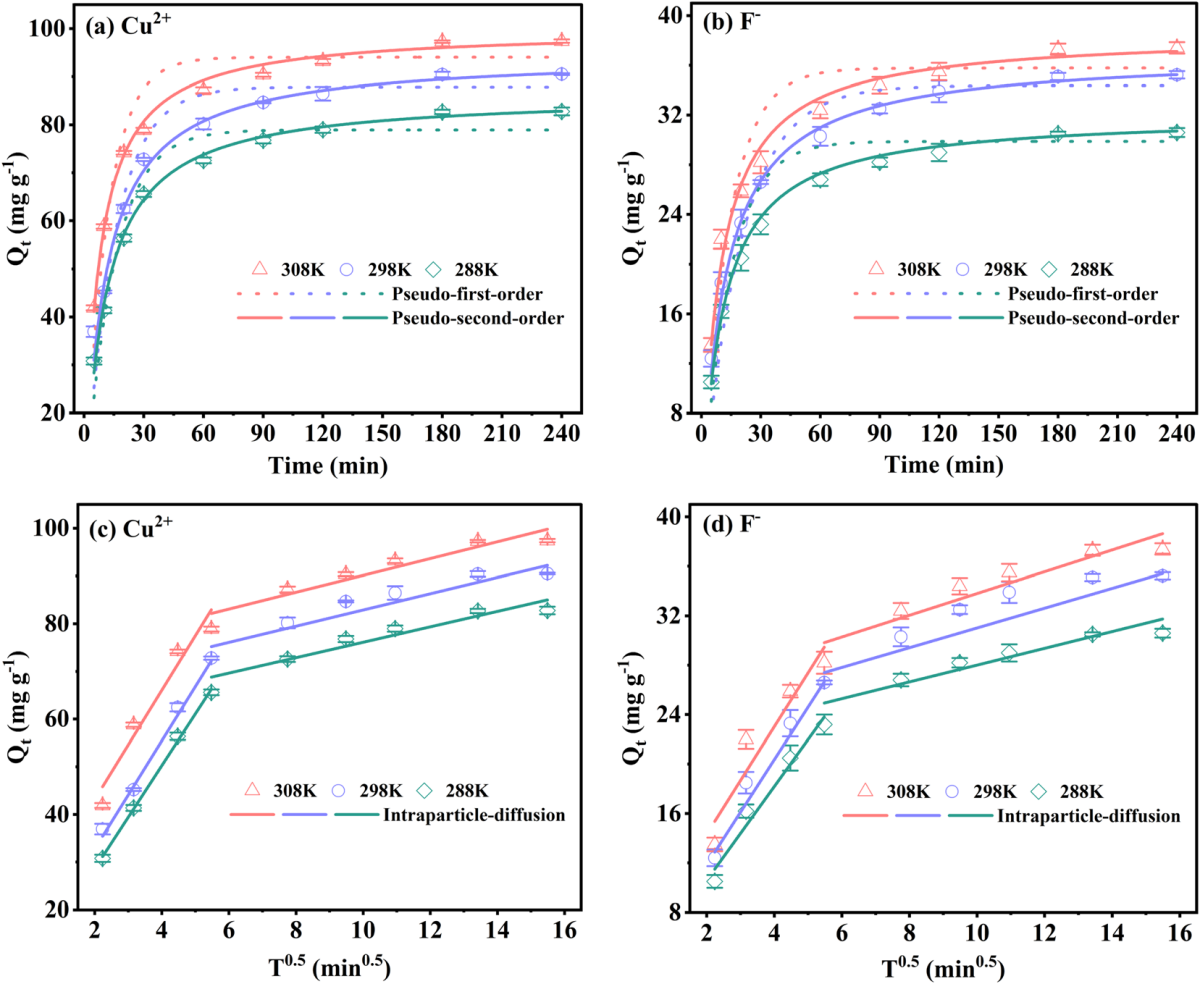
Pseudo-first-order and pseudo-second-order kinetics of Cu^2+^ (a) and F^−^ (b); intraparticle-diffusion model of Cu^2+^ (c) and F^−^ (d).

Fig. 7(a) and (c) show the pseudo-first-order and pseudo-second-order kinetic model curves of Cu^2+^ and F^−^ adsorption at 288 K, 298 K, and 308 K, respectively. The adsorption rates for Cu^2+^ and F^−^ increase at higher temperatures, suggesting that temperature reinforces Cu^2+^ and F^−^ adsorption by FKBC. In addition, the statistical parameters of kinetic models are demonstrated in Table 2. Compared to the pseudo-first-order kinetics model, the pseudo-second-order kinetics model better fits Cu^2+^ (*R*^2^ = 0.989–0.997) and F^−^ (*R*^2^ = 0.988–0.998) adsorption rate at each temperature. The Cu^2+^ and F^−^ adsorption capacity calculated (*Q*_e,cal_) by pseudo-second-order kinetics is closer to the experimental data (*Q*_e,exp_), indicating that the adsorption behavior of Cu^2+^ and F^−^ is more suitable to Pseudo-second-order kinetics and mainly controlled by chemisorption.^4,38^

**Table 2:** Adsorption kinetics parameters for Cu^2+^ and F^−^ adsorption at different temperatures

Parameters	Cu^2+^			F^−^		
	288 K	298 K	308 K	288 K	298 K	308 K
*Q*_e,exp_ (mg g^−1^)	82.8	90.5	97.4	30.6	35.2	37.4

**Pseudo-first-order**						
*Q*_e,cal1_ (mg g^−1^)	78.1	85.7	91.3	28.7	32.8	34.7
*k*_1_ (min^−1^)	0.072	0.075	0.099	0.070	0.072	0.082
*R*_1_^2^	0.954	0.939	0.930	0.949	0.940	0.918

**Pseudo-second-order**						
*Q*_e,cal_^2^ (mg g^−1^)	85.5	93.4	98.5	31.5	35.9	37.8
*k*_2_ × 10^3^ (g mg^−1^ min^−1^)	1.09	1.16	1.48	2.14	2.78	3.06
*R*_2_^2^	0.996	0.989	0.997	0.998	0.996	0.988

**Intraparticle-diffusion**						
*k*_3I_ (mg g^−1^ min^−0.5^)	10.83	11.38	11.42	3.82	4.27	4.30
*C*_1_ (mg g^−1^)	7.00	10.69	19.63	2.95	3.81	5.92
*R*_3I_^2^	0.998	0.993	0.949	0.967	0.975	0.900
*K*_3II_ (mg g^−1^ min^−0.5^)	1.64	1.70	1.75	0.68	0.80	0.85
*C*_2_ (mg g^−1^)	59.16	66.24	72.21	20.84	23.62	25.17
*R*_3II_^2^	0.910	0.904	0.912	0.879	0.898	0.882

Fig. 7(b) and (d) show the intraparticle-diffusion model for Cu^2+^ and F^−^ adsorption on FKBC, respectively. Both curves display two linear portions at each temperature, indicating that the adsorption process is divided into two diffusion stages.^51^ The intraparticle-diffusion rate constant of the two stages is shown in Table 2. Cu^2+^ and F^−^ may have first diffused on the FKBC surface at a high rate and then diffused into micropores at a lower rate.^37^ The linear curves do not traverse the origin, revealing that the adsorption rate is determined by intraparticle-dispersion and physical and chemical processes.^52^

### 3.4 Adsorption isotherms

To evaluate the adsorption capacity of Cu^2+^ and F^−^ by FKBC, the experimental data are fitted using the Langmuir, Freundlich, and Temkin isotherm models. Langmuir isotherm model indicates that the adsorption process is monolayer chemisorption on a homogeneous surface.^19,21^ At the same time, the Freundlich isotherm model describes the multi-layer physisorption on heterogeneous surfaces.^19^ The Temkin isotherm model is appropriate for chemisorption and assumes that adsorption energy decreases linearly with the surface coverage.^14^

Langmuir isotherm (eqn (7)), Freundlich isotherm (eqn (8)) and Temkin isotherm (eqn (9)) models are expressed as follows:7
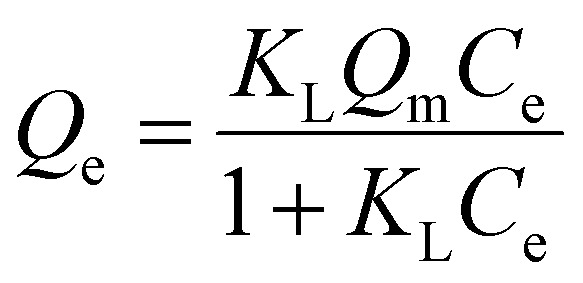

8 *Q*_e_ = *K*_F_*C*_e_^1/*n*^

9 *Q*_e_ = *B* ln *K*_T_ + *B* ln *C*_e_

where *K*_L_ (L mg^−1^) is the Langmuir constant related to the adsorption sites, *Q*_m_ (mg g^−1^) is the maximum adsorption capacity, *K*_F_ [(mg g^−1^) (mg L^−1^)^*n*^] and *n* are Freundlich constants related to adsorption capacity and adsorption density, respectively. *B* (J mol^−1^) is the heat of the adsorption constant, and *K*_T_ (L g^−1^) is the equilibrium binding constant related to the Temkin model.

**Fig. 8:**
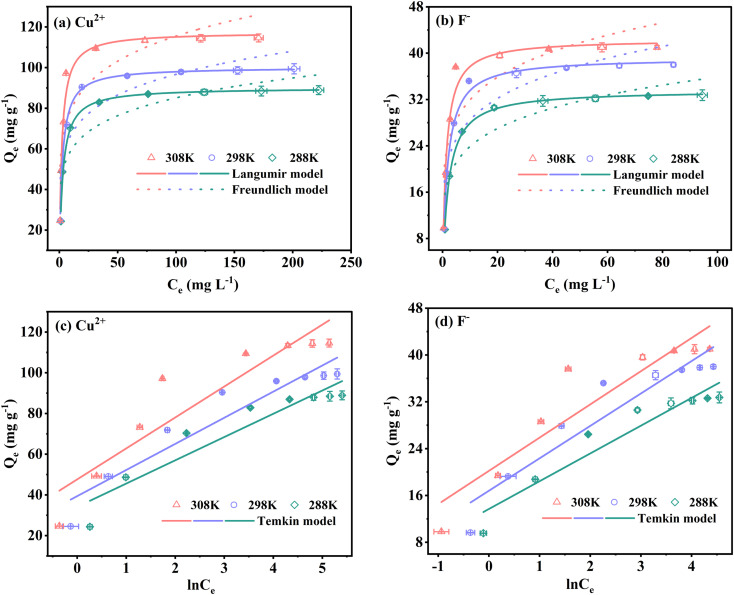
Langmuir and Freundlich isotherm models of Cu^2+^ (a) and F^−^ (b); Temkin isotherm model of Cu^2+^ (c) and F^−^ (d).

Fig. 8(a) and (c) depict that the Langmuir isotherm model curves are better fitted than the Freundlich model at each temperature. Based on the isotherm parameters, the Langmuir isotherm model exhibits higher regression coefficients for Cu^2+^ and F^−^ than those of the Freundlich model, respectively (Table 3). The above results describe that the Langmuir isotherm model describes the Cu^2+^ and F^−^ adsorption behavior, illustrating that the monolayer adsorption of Cu^2+^ and F^−^ mainly is on the homogeneous surface of FKBC, and adsorption is mostly chemisorption.^53,54^ At the same time, the separation factor *R*_L_ (*R*_L_ = 1/(1 + *K*_L_ × *C*_0_)) demonstrates the applicability of the adsorption experiment.^55^ However, 0 < *R*_L_ ≤ 1 and *R*_L_ > 1 indicate that the adsorption is favorable and unfavorable, respectively.^5^ The *R*_L_ values are all within the range of 0–1 at each temperature, indicating the favorable nature of the Cu^2+^ and F^−^ adsorption using FKBC (Table 3).

**Table 3:** Adsorption isotherm parameters for Cu^2+^ and F^−^ adsorption at different temperatures

Parameters	Cu^2+^			F^−^		
	288 K	298 K	308 K	288 K	298 K	308 K
*Q*_e,exp_ (mg g^−1^)	82.8	90.5	97.4	30.6	35.2	37.4

**Langmuir isotherm**						
*Q*_m,cal_ (mg g^−1^)	90.1	100.2	117.4	33.6	39.2	42.4
*K*_L_ (L mg^−1^)	0.37	0.43	0.50	0.49	0.59	0.80
*R*_L_	0.013	0.011	0.009	0.025	0.021	0.015
*R*_1_^2^	0.990	0.994	0.982	0.998	0.990	0.981

**Freundlich isotherm**						
*K*_F_ (mg g^−1^) (mg L^−1^)^n^	41.27	46.15	54.50	15.89	18.89	21.68
*n* (g mg^−1^ min^−1^)	6.36	6.22	6.13	5.66	5.62	5.94
*R*_2_^2^	0.820	0.827	0.783	0.839	0.809	0.786

**Temkin isotherm**						
*B* (J mol^−1^)	11.43	12.87	15.23	4.75	5.55	5.71
*K*_T_ (g L^−1^)	19.75	21.06	22.65	17.78	20.38	33.97
*R*_3_^2^	0.889	0.901	0.862	0.910	0.887	0.867

Fig. 8(b), (d), and Table 3 show the Temkin isotherm model fitting lines and related coefficients. The heat of the adsorption constant (*B*) of the Temkin model increases with increasing temperature, indicating the endothermic adsorption process.^56^ The investigated parameters also reveal that the maximum adsorption capacities (*Q*_m_) for Cu^2+^ and F^−^ based on the Langmuir model increase by 30.4% and 26.2% when the temperature increases from 288 K to 308 K, respectively. These results suggest that increasing temperature is conducive to reaction progression in Cu^2+^ and F^−^ adsorption by FKBC, consistent with the Temkin model's investigation.

Some previously reported adsorbents with Cu^2+^ and F^−^ adsorption capabilities are compared with FKBC (Table 4). FKBC can effectively adsorb Cu^2+^ and F^−^, an advantage previously studied adsorbents do not possess.

**Table 4:** Comparison of FKBC adsorption capacity for Cu^2+^ and F^−^ with previously reported adsorbents

Adsorbent	ion	pH	*T* (K)	*Q*_m_ (mg g^−1^)	Reference
Ferromanganese oxide–biochar	Cu^2+^	5	308	65.9	5
BC-LDHs	Cu^2+^	5	298	85.47	53
Salt-based biochar	Cu^2+^	5–6	308	187.76	57
Modified bamboo charcoal	Cu^2+^	5	318	40.65	29
FeOOH/TOCN powder	F^−^	7	313	19	37
Granular ferric hydroxide	F^−^	6–7	298	7.0	3
Ca-doped ferrihydrite	F^−^	5.75	298 ± 2	53.21	33
Ternary metal oxide	F^−^	7	298	63.05	58
FKBC	Cu^2+^	5	308	117.4	This work
	F^−^	7	308	42.4	

### 3.5 Adsorption thermodynamics

Thermodynamic parameters of the adsorption process involving changes in Gibbs free energy (Δ*G*^0^), enthalpy (Δ*H*^0^), and entropy (Δ*S*^0^) are calculated to examine the spontaneity and feasibility of Cu^2+^ and F^−^ adsorption using the following equations:^59^

10 Δ*G*^0^ = −*RT* ln *K*_C_

11
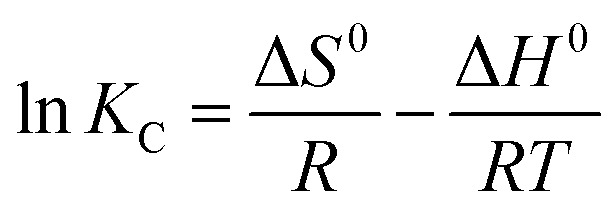

12 *K*_C_ = *M* × 55.5 × 1000 × *K*_L_

where *R* [8.314 (J mol^−1^ K^−1^)] is the universal gas constant, *T* (K) is the temperature in Kelvin, *K*_C_ is an equilibrium coefficient related to Langmuir constant *K*_L_ (L mg^−1^), *M* (g mol^−1^) is the adsorbate molar mass, and 55.5 corresponds to the solvent molar concentration (mol L^−1^).^3^

The Δ*H*^0^ and Δ*S*^0^ values are calculated from the slope and intercept of straight lines (Fig. S3†), while Δ*G*^0^ values are obtained from eqn (10). The Δ*G*^0^, Δ*H*^0^, and Δ*S*^0^ values are listed in Table 5.

**Table 5:** Thermodynamic factors for Cu^2+^ and F^−^ adsorption on FKBC at different temperatures

Ion	Temperature (K)	Thermodynamic parameters		
		Δ*G*^0^ (kJ mol^−1^)	Δ*S*^0^ (J mol^−1^ K^−1^)	Δ*H*^0^ (kJ mol^−1^)
Cu^2+^	288	−33.69	156.39	11.35
	298	−35.26		
	308	−36.82		
F^−^	288	−31.44	172.52	18.24
	298	−33.17		
	308	−34.89		

Δ*G*^0^ values are negative at each temperature, suggesting that the Cu^2+^ and F^−^ adsorption by FKBC are all spontaneous and follow the chemisorption process.^4,37^ In addition, the Δ*G*^0^ values are more negative with increasing temperature, demonstrating that a higher temperature is thermodynamically favorable to Cu^2+^ and F^−^ adsorption.^32,35^

Δ*S*^0^ values are greater than zero, suggesting increased randomness at the solid/liquid interface for Cu^2+^ and F^−^ adsorption.^14,49^ Δ*H*^0^ values also are positive, corroborating that the adsorption is endothermic, agreeing well with the adsorption above isotherm analysis.^3,32^

### 3.6 Adsorption mechanism

**Fig. 9:**
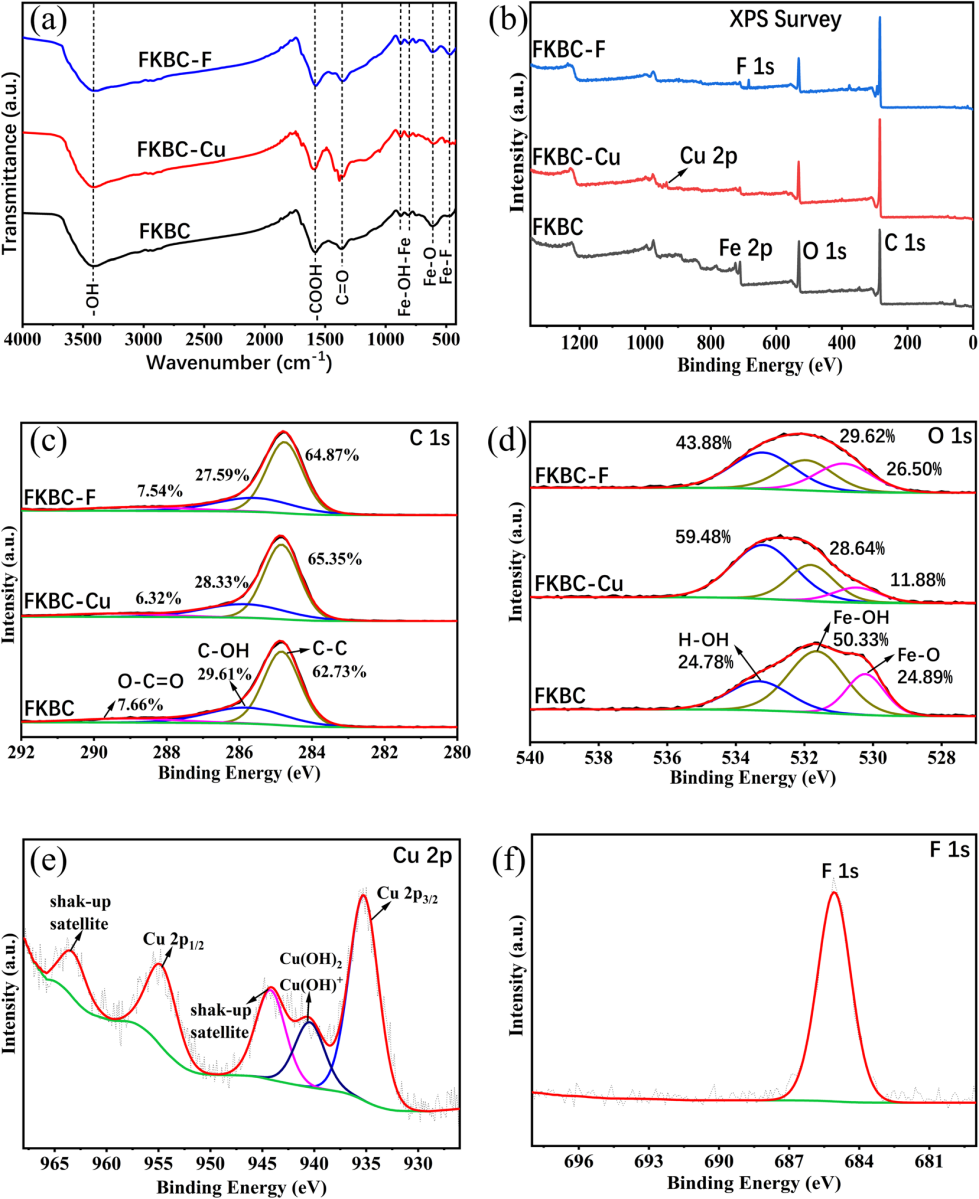
Spectra of FKBC before and after Cu^2+^ and F^−^ adsorption. (a) FTIR; (b) full scan XPS; (c) XPS C1s of FKBC after Cu^2+^ and F^−^ adsorption; (d) XPS O1s of FKBC after Cu^2+^ and F^−^ adsorption; (e) XPS Cu2p of FKBC after Cu^2+^ adsorption; (f) XPS F1s of FKBC after F^−^ adsorption.

Functional group changes in FKBC before and after Cu^2+^ and F^−^ adsorption are shown in the FTIR spectrum (Fig. 9(a)). The peak at 3414 cm^−1^ is the bending and stretching of –OH groups on FKBC.^5^ The peaks at 1580 cm^−1^ and 1364 cm^−1^ are respectively related to the tension of –COOH and symmetric stretching of the carboxyl group, and peaks at 877 cm^−1^ and 815 cm^−1^ are attributed to the extension and in-plane bending of surface hydroxyl groups on α-FeOOH.^52^ After Cu^2+^ and F^−^ adsorption, these peaks underwent corresponding changes. During Cu^2+^ adsorption, the oxygen atoms in –OH and –COOH groups share their non-bonding electron pairs with Cu^2+^, thereby reacting to form surface complexes (such as COO–Cu, Fe–O–Cu, C–O–Cu) and substituting H in these groups.^5,50^ During F^−^ adsorption, the –OH is replaced by F^−^, or hydrogen bonding occurs between F^−^.^60^ In addition, the peak at 613 cm^−1^ is attributed to Fe–O bending vibrations.^52^ After Cu^2+^ adsorption, the peak weakens, preliminarily indicating that Cu may replace some Fe in α-FeOOH. Moreover, after the F^−^ adsorption, the peak at 613 cm^−1^ changes slightly, and a new absorption peak appears at 467 cm^−1^, possibly due to Fe–F formation. Overall, preliminary FTIR analysis shows that the oxygen-containing functional groups on the FKBC surface are mainly consumed during Cu^2+^ and F^−^ adsorption, leading to the movement of characteristic O–H bands.^5^

To further explore the adsorption mechanism of Cu^2+^ and F^−^, the high-resolution XPS spectra obtained are shown in Fig. 9(b)–(e). XPS full spectrum showed that after FKBC adsorption, Cu and F element signals are detected near 935.1 and 684.5 eV, respectively, confirming the successful Cu^2+^ and F^−^ adsorption (Fig. 9(b)). This is consistent with the EDS element analysis results in Fig. 1(c) and (d).

Fig. 9(c) shows the core energy level spectrum of C1s, divided into three peaks at 288.9, 286.1, and 284.8 eV, generated by the O–C=O bond, C–OH bond, and C–C bond, respectively.^57^ After Cu^2+^ and F^−^ adsorption, the C–OH bonds and O–C=O bonds relative content decreased; however, the reduction is insignificant. This indicates that the oxygen-containing functional groups connected to the C are not the main Cu^2+^ and F^−^ adsorption participants.

The O1s spectrum in Fig. 9(d) is also divided into three peaks at 533.4 eV, 531.5 eV, and 530.3 eV, corresponding to O_H_2_O_ (adsorbed water molecules), O_ad_ (chemically adsorbed oxygen), and O_latt_ (lattice oxygen), generated by H–O–H, Fe–OH and Fe–O bonds, respectively.^31,35^ Before adsorption, the relative Fe–OH content is 50.33%, significantly higher than Fe–O (24.89%), confirming that FKBC contains abundant hydroxyl radicals due to the existence of α-FeOOH.^38^ After Cu^2+^ adsorption, the relative contents of Fe–OH and Fe–O decreased significantly, confirming that Cu^2+^ replaces the hydrogen in hydroxyl groups to form a complex structure with oxygen and exchanges some Fe in α-FeOOH and then forms the O–Cu bond. This conclusion is consistent with FTIR analysis and is confirmed by Cu2p spectroscopy. After F^−^ adsorption, there is a significant decrease in Fe–OH, and the relative content of Fe–O remained unchanged, indicating that the hydroxyl groups in α-FeOOH are exchanged by F^−^, while Fe is not involved in the adsorption process.

The Cu2p spectrum shows that the binding energy exhibits typical Cu2p_3/2_ and Cu2p_1/2_ characteristic peaks at 935 and 955 eV and shake-up satellite peaks at 963.4 and 943.8 eV, suggesting that Cu^2+^ is adsorbed in a bivalent state and O–Cu bonds are formed (Fig. 9(e)).^57^ In addition, a peak near 940 eV may be related to Cu(OH)_2_ or Cu(OH)^+^, implying the existence of micro-precipitation on the adsorbent surface,^61^ confirming the influence of pH on Cu^2+^ adsorption. After the F^−^ adsorption, the spectrum shows a typical F1s characteristic peak, and the binding energy is between 684–685 eV, indicating that the adsorbed F^−^ mainly existed as inorganic fluoride (Fig. 9(f)). EDS mapping results after adsorption reveal that the Cu and F elements almost overlap with the sediment area located on FKBC, suggesting that the α-FeOOH on the FKBC surface mainly contributes to Cu^2+^ and F^−^ adsorption (Fig. S1(d–g), S2(d–g)†).

**Fig. 10:**
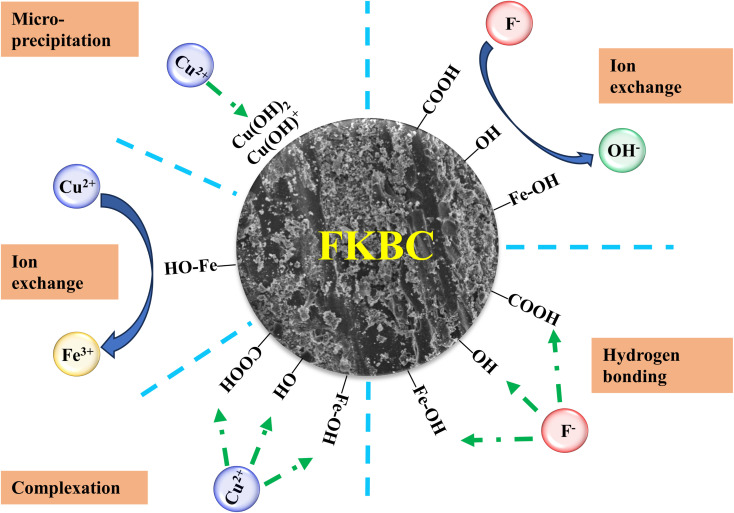
Proposed Cu^2+^ and F^−^ adsorption mechanisms by FKBC.

In summary, the Cu^2+^ adsorption mechanism mainly relies on ion exchange, complexation, and micro-precipitation, while F^−^ mainly relies on ion exchange and hydrogen bonding (Fig. 10). However, other mechanisms cannot be ruled out, and further research is needed.

### 3.7 Selective adsorption

**Fig. 11:**
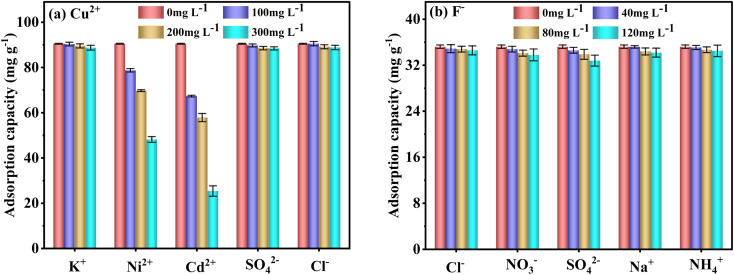
Effect of coexisting ions on the selective Cu^2+^ (a) and F^−^ (b) adsorption by FKBC.

To evaluate the FKBC selective adsorption performance, the effect of coexisting ions at different concentrations on Cu^2+^ and F^−^ adsorption capacity and efficiency is examined. The order of coexisting ions affecting Cu^2+^ adsorption is Cd^2+^ > Ni^2+^ > K^+^ ≈ SO_4_^2−^ ≈ Cl^−^ (Fig. 11(a)). Additionally, the coexisting K^+^, SO_4_^2−^, and Cl^−^ ions have little effect on Cu^2+^ adsorption since the active adsorption sites on the FKBC surface form a stable complex with Cu^2+^; thus, selective adsorption is high for Cu^2+^. Also, Cd^2+^ has a greater impact on the Cu^2+^ selective adsorption than Ni^2+^, possibly due to its larger ionic radius (Cd^2+^ (0.095 nm) > Cu^2+^ (0.073 nm) > Ni^2+^ (0.069 nm)),^62^ exhibiting a competitive effect on Cu^2+^ adsorption process. However, comparing the adsorption capacities and efficiencies of Ni^2+^ and Cd^2+^ with that of Cu^2+^ (Table S4†), FKBC shows higher selectivity for Cu^2+^ at low Ni^2+^ and Cd^2+^ concentrations, attributed to the hydration. Since the hydration ion radius of Cu^2+^ (0.404 nm) is smaller than that of Cd^2+^ (0.426 nm) and Ni^2+^ (0.419 nm),^62^ thus, their contact is easy with the FKBC surface and enters the internal pores.

Fig. 11(b) indicates that the coexisting Cl^−^, NO_3_^−^, SO_4_^2−^, Na^+^, and NH_4_^+^ have little effect on F^−^ adsorption. The adsorption capacities and efficiencies of coexisting ions are far below that of F^−^ by FKBC (Table S5†), illustrating that the FKBC surface sites have selective F^−^-adsorption due to hydrogen bonds with F^−^.

### 3.8 Reusability assessment

**Fig. 12:**
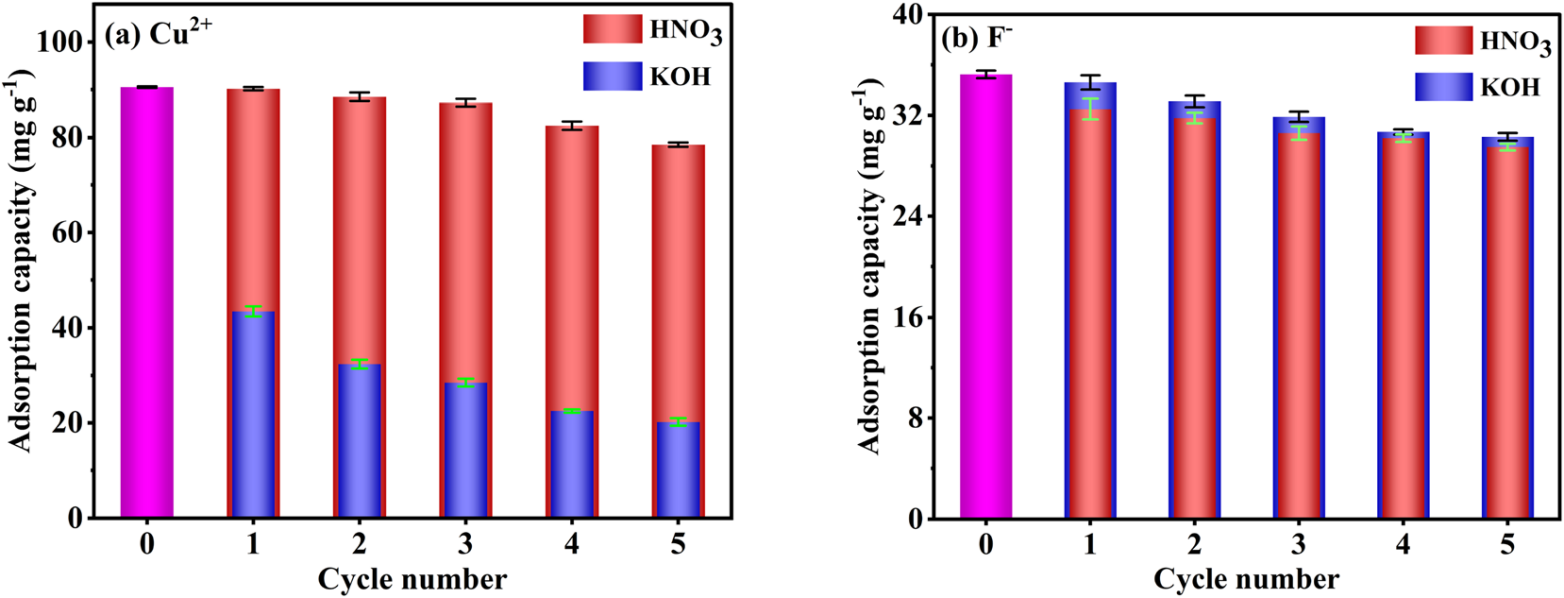
Reusability of FKBC for Cu^2+^ (a) and F^−^ (b) after desorption-regeneration using HNO_3_ and KOH, respectively.

Adsorption–desorption cycle experiment results are presented in Fig. 12. The adsorption capacities of F^−^ slowly decrease with increasing cycle number and remain at 29.5 and 30.3 mg g^−1^ after five cycles with HNO_3_ and KOH desorption, respectively. In addition, there is no significant difference in F^−^ adsorption capacities of FKBC regenerated by HNO_3_ and KOH in the same cycle number, suggesting the feasibility of using acid or alkali to desorb F^−^. However, the adsorption capacities of Cu^2+^ after desorption with HNO_3_ (90.2–78.5 mg g^−1^) are substantially higher after desorption with KOH (43.5–20.2 mg g^−1^). Based on the effect of pH, some adsorption sites on the adsorbent surface are inevitably occupied by precipitated flocs when using KOH to desorb Cu^2+^. Overall, the FKBC is a potentially promising adsorbent for Cu^2+^ and F^−^ due to its high reusability, while using alkali for Cu^2+^ desorption should be avoided.

## 4. Conclusion

A novel adsorbent, FKBC, is successfully prepared for Cu^2+^ and F^−^ adsorption. The characterization results indicate that FKBC has a mesoporous structure after KOH modification, and the surface loading α-FeOOH has also been successful. Compared with raw BC, FKBC exhibits a larger specific surface area, pore volume, and smaller pore size, providing more adsorption sites and favorable conditions for Cu^2+^ and F^−^ adsorption. Its adsorption effect for Cu^2+^ and F^−^ heavily depends on the solution pH, contact time, dosage, Cu^2+^, and F^−^ initial concentration. The FKBC adsorption kinetics shows that the pseudo-second-order kinetics model better fits the adsorption rate at each temperature. Based on the well-fitted Langmuir isotherm model, the maximum monolayer adsorption capacity of FKBC for Cu^2+^ and F^−^ at 308 K is estimated at 117.4 mg g^−1^ and 42.4 mg g^−1^, respectively. Thermodynamic equations prove that increasing temperature can promote Cu^2+^ and F^−^ adsorption by FKBC. The adsorption mechanism suggests that the process mainly involves chemical adsorption, and the α-FeOOH on the FKBC surface mainly contributes to Cu^2+^ and F^−^ adsorption. In addition, the selective adsorption results indicate that FKBC is a selective adsorbent for Cu^2+^ and F^−^. The adsorption–desorption results also prove that FKBC has high recycling value. Overall, FKBC can potentially adsorb both Cu^2+^ and F^−^ effectively.

## Data availability

The data used and analyzed during the current study are available from the corresponding author upon reasonable request.

## Author contributions

Conceptualization, investigation, writing-original draft, funding acquisition, Wei Yang; investigation, Lei Zhang; conceptualization, methodology, Meng Li; data curation, Ting Zhang; project administration, Yue Liu; resources, Juan Liu.

## Conflicts of interest

The authors declare no competing interests.

## Supplementary Material

RA-013-D3RA05315F-s001
