# Supplementary material for: KOH-modified bamboo charcoal loaded with α-FeOOH for efficient adsorption of copper and fluoride ions from aqueous solution
Source: RSC Adv. 2023 Oct 16;13(43):30176–89. doi: 10.1039/d3ra05315f (PMC10577395; doi:10.1039/d3ra05315f)
Supplement: RA-013-D3RA05315F-s001 [file RA-013-D3RA05315F-s001.pdf]

Electronic Supplementary Material (ESI) for RSC Advances.
This journal is © The Royal Society of Chemistry 2023

Supplementary information

KOH-modified bamboo charcoal loaded with α-FeOOH for efficient adsorption of copper and fluoride ions from aqueous solution

Wei Yang, [*a] Lei Zhang, [b] Meng Li, [c] Ting Zhang, [a] Yue Liu [a] and Juan Liu [a]

[a] School of Environmental Science and Engineering, Hubei Polytechnic University, Huangshi 435003, Hubei, China. E-mail: yw198909@163.com

[b] MWR Standard & Quality Control Research Institute, Hangzhou 310024, Zhejiang, China.

[c] School of Civil Engineering and Architecture, Wuhan University of Technology, Wuhan 430070, Hubei, China

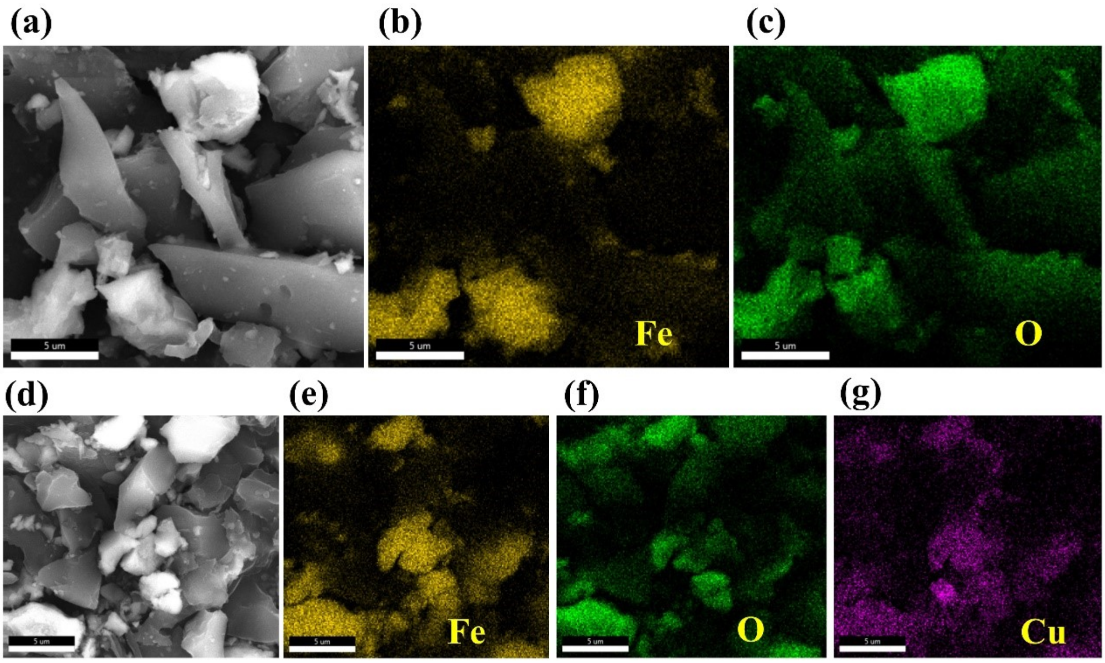

Fig. S1 SEM images of FKBC before (a), and after (d) Cu^{2+} adsorption; EDS elemental mappings of Fe (b), O (c) before, and Fe (e), O (f), Cu (g) after Cu^{2+} adsorption.

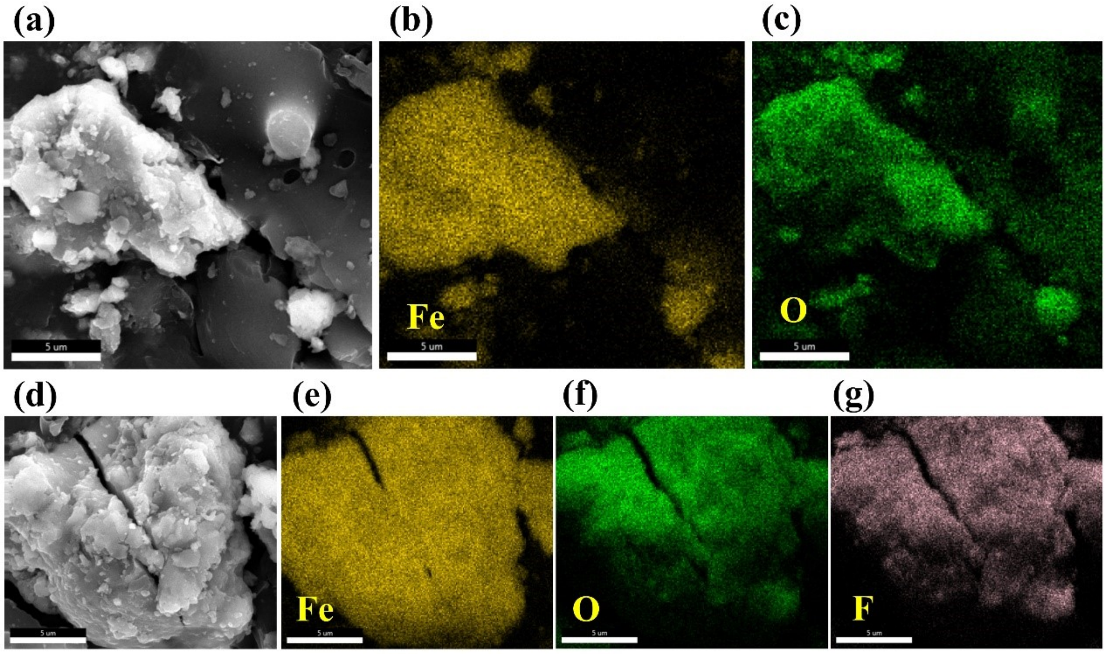

Fig. S2 SEM images of FKBC before (a), and after (d) F^- adsorption; EDS elemental mappings of Fe (b), O (c) before, and Fe (e), O (f), F (g) after F^- adsorption.

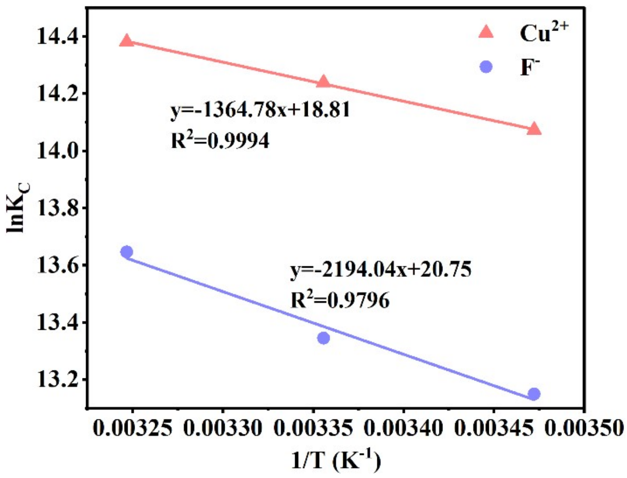

Fig. S3 Thermodynamic analysis of Cu^{2+} and F^- adsorption by FKBC.

Table S1 Batch experimental conditions for Cu^{2+} and F^- adsorption by FKBC.

Influencing factor	Ion	Volume (mL)	pH	Time (min)	Dosage (g)	Concentration (mg L^{-1})	Temperature (K)
pH	Cu^{2+}	100	2-6	240	0.2	200	298
	F^-	100	2-10	240	0.2	80	298
Contact time	Cu^{2+}	100	5	5-240	0.2	200	298
	F^-	100	7	5-240	0.2	80	298
Adsorbent dose	Cu^{2+}	100	5	240	0.05-0.4	200	298
	F^-	100	7	240	0.05-0.4	80	298
Initial concentration	Cu^{2+}	100	5	240	0.2	50-400	298
	F^-	100	7	240	0.2	20-160	298
Kinetics	Cu^{2+}	100	5	5-240	0.2	200	288-308
	F^-	100	7	5-240	0.2	80	288-308
Isotherms	Cu^{2+}	100	5	240	0.2	50-400	288-308
	F^-	100	7	240	0.2	20-160	288-308
Thermodynamics	Cu^{2+}	100	5	240	0.2	50-400	288-308
	F^-	100	7	240	0.2	20-160	288-308

Table S2 Experimental conditions on the effect of coexisting ions for selective Cu^{2+} and F^- adsorption by FKBC.

Ion	Volume (mL)	Solution pH	Initial concentration (mg L^{-1})	Contact time（min）	Dosage (g)	Coexisting ions	Coexisting ions concentration (mg L^{-1})	Temperature (K)
Cu^{2+}	100	5	200	240	0.2	K^+<br>Ni^{2+}<br>Cd^{2+}<br>Cl^-<br>SO_4^{2-}	0、100、200、300	298K
F^-	100	7	80	240	0.2	Cl^-<br>NO_3^-<br>SO_4^{2-}<br>Na^+<br>NH_4^+	0、40、80、120	298K

Table S3 Experimental conditions for Cu^{2+} and F^- adsorption-desorption.

Ion	Volume (mL)	Solution pH	Initial concentration (mg L^{-1})	Adsorbent dosage (g)	Contact time (min)	Desorption reagent	Desorption time (min)	Temperature (K)
Cu^{2+}	100	5	200	0.2	240	HNO_3 (1mol L^{-1})	60	298K
	100	5	200	0.2	240	KOH (1mol L^{-1})	60	298K
F^-	100	7	80	0.2	240	HNO_3 (1mol L^{-1})	60	298K
	100	7	80	0.2	240	KOH (1mol L^{-1})	60	298K

Table S4 Experimental results on the effect of coexisting ions for selective Cu^{2+} adsorption by FKBC.

Coexisting Ion	Ion concentration (mg L^{-1})	Adsorption capacity (mg g^{-1})	Adsorption efficiency (%)	Cu^{2+}-adsorption capacity (mg g^{-1})	Cu^{2+}-adsorption efficiency (%)
	100	5.1	10.2	90.4	90.4
K^+	200	8.9	8.9	89.6	89.6
	300	12.8	8.5	88.8	88.8
	100	9.6	19.2	78.8	78.8
Ni^{2+}	200	22.6	22.6	69.8	69.8
	300	55.7	37.1	48.2	48.2
	100	12.6	25.2	67.4	67.4
Cd^{2+}	200	40.2	40.2	57.9	57.9
	300	96.8	64.5	25.4	25.4
	100	8.4	16.8	89.8	89.8
SO_4^{2-}	200	15.6	15.6	88.6	88.6
	300	21.5	14.3	88.5	88.5
	100	7.6	15.2	90.6	90.6
Cl^-	200	14.2	14.2	89.2	89.2
	300	18.1	12.1	88.9	88.9

(Ni^{2+}, Cd^{2+} concentrations were determined by atomic absorption spectroscopy (AAS)； K^+, Cl^-, SO_4^{2-} were determined by ion chromatography (IC)).

Table S5 Experimental results on the effect of coexisting ions for selective F^- adsorption by FKBC.

Coexisting Ion	Ion concentration (mg L^{-1})	Adsorption capacity (mg g^{-1})	Adsorption efficiency (%)	F^--adsorption capacity (mg g^{-1})	F^--adsorption efficiency (%)
	40	1.5	7.5	34.9	87.3
Cl^-	80	2.2	5.5	34.8	87.0
	120	4.4	7.3	34.6	86.5
	40	1.6	8.0	34.8	87.0
NO_3^-	80	3.3	8.3	34.1	85.3
	120	3.7	6.2	33.8	84.5
	40	2.8	14.0	34.6	86.5
SO_4^{2-}	80	4.6	11.5	33.9	84.8
	120	5.8	9.7	32.8	82.0
	40	2.3	11.5	35.2	88.0
Na^+	80	2.4	6.0	34.4	86.0
	120	3.1	5.2	34.2	85.5
	40	2.4	12.0	35.1	87.8
NH_4^+	80	2.9	7.3	34.7	86.8
	120	4.2	7.0	34.5	86.3

(Na^+, NH_4^+, Cl^-, NO_3^-, SO_4^{2-} were determined by ion chromatography (IC)).
